# KAT5-mediated SOX4 acetylation orchestrates chromatin remodeling during myoblast differentiation

**DOI:** 10.1038/cddis.2015.190

**Published:** 2015-08-20

**Authors:** S-M Jang, J-W Kim, C-H Kim, J-H An, A Johnson, P I Song, S Rhee, K-H Choi

**Affiliations:** 1 Department of Life Science, Chung-Ang University, Seoul 156-756, Korea; 2 Department of Dermatology, University of Arkansas for Medical Sciences, Little Rock, AR 72205, USA; 3 Department of Dermatology, University of Colorado Denver Anschutz Medical Campus, Aurora, CO 80045, USA

## Abstract

Transcription factor SOX4 has been implicated in skeletal myoblast differentiation through the regulation of *Cald1* gene expression; however, the detailed molecular mechanism underlying this process is largely unknown. Here, we demonstrate that SOX4 acetylation at lysine 95 by KAT5 (also known as Tip60) is essential for *Cald1* promoter activity at the onset of C2C12 myoblast differentiation. KAT5 chromodomain was found to facilitate SOX4 recruitment to the *Cald1* promoter, which is involved in chromatin remodeling at the promoter. Chromatin occupancy analysis of SOX4, KAT5, and HDAC1 indicated that the expression of putative SOX4 target genes during C2C12 myoblast differentiation is specifically regulated by the molecular switching of the co-activator KAT5 and the co-repressor HDAC1 on SOX4 transcriptional activation.

Myoblast differentiation requires a series of orchestrated steps including cell cycle withdrawal, alignment, and the fusion of mononucleated myoblasts to form multinucleated myotubes.^1, 2, 3, 4^ Numerous transcription factors (TFs) such as MyoD, Myf5, myogenin, SOX4, and other undefined intrinsic factors respond to extrinsic signaling during the differentiation.^5, 6, 7^ TFs initiate and execute myoblast differentiation together with histone-modifying enzymes.^8, 9^ MyoD-centered transcriptional regulation via molecular switching between repressors and activators is well studied during myoblast differentiation.^5^ In undifferentiated myoblasts, HDAC1 interacts with MyoD. This interaction maintains chromatin in a compact structure by preventing histone hyperacetylation at the response elements of muscle genes, which inhibits MyoD loading on target genes in undifferentiated myoblasts.^1, 10^ Differentiation cues promote HDAC1 downregulation and dissociation from MyoD, which enables free MyoD to interact with coactivators, such as p300/CBP and PCAF, resulting in MyoD acetylation and muscle gene expression.^11, 12, 13, 14^ Thus, protein–protein interactions and posttranslational modifications (PTMs) likely contribute to the temporal regulation of numerous muscle-specific TFs during myoblast differentiation.

Changes in chromatin organization regulate gene expression during tissue differentiation.^15^ Biochemical modifications of histones, such as acetylation or methylation of lysine residues, directly influence chromatin structure.^8, 9^ Thus, it is plausible that histone PTMs serve as a ‘histone code' to recruit effector molecules to chromatin, and this process ultimately determines the functional outcome of certain signals.^16, 17, 18^ Several protein families have been identified as histone code-recognizing factors. Code reader-mediated protein–chromatin interaction changes in histone modifications, and chromatin remodeling enables the recruitment of multi-protein complexes to active loci, leading to gene transcription. Therefore, the functional combination of histone code reader proteins and TFs serves as a crucial paradigm for understanding the mechanisms underlying tissue- or cell type-specific gene expression and cellular differentiation. Lysine acetyltransferase 5 (KAT5), which was originally named TIP60 (HIV-1 Tat Interactive Protein, 60 kDa), induces HIV-1 Tat transcriptional activation.^19^ The presence of a MYST (MOZ, Ybf2/Sas3, SAS2, and TIP60) domain classifies KAT5 as a member of the MYST family of histone acetyltransferases (HATs), which participate in various cellular processes including transcriptional regulation, development, apoptosis, and DNA damage repair.^20, 21, 22, 23, 24, 25, 26^ Through its HAT activity, KAT5 catalyzes the acetylation of core histones (H2A, H3, and H4) and several non-histone proteins including the p53 and MYC TFs.^27, 28, 29, 30, 31, 32^ In addition to a HAT domain, KAT5 has a chromodomain that enables interaction with methylated histones, and thereby it has potential as a histone code reader.^18^

We have previously shown that SOX4 as a primary TF regulates *Cald1* expression during C2C12 myoblast differentiation.^7^ However, the molecular mechanism underlying the temporal regulation of SOX4 transcriptional activation during differentiating myoblasts is largely unknown. We found in this study that SOX4 TF was specifically acetylated by KAT5 under differentiation conditions. Transcriptional activity and protein loading of SOX4 to its target gene promoter regions were affected by acetylation status and histone code reading by the KAT5 chromodomain. Our results suggest that KAT5 mediates functional roles between chromatin remodeling and PTM of SOX4 during myoblast differentiation.

## Results

### SOX4 acetylation is specifically regulated by KAT5 during myoblast differentiation

**Figure 1:**
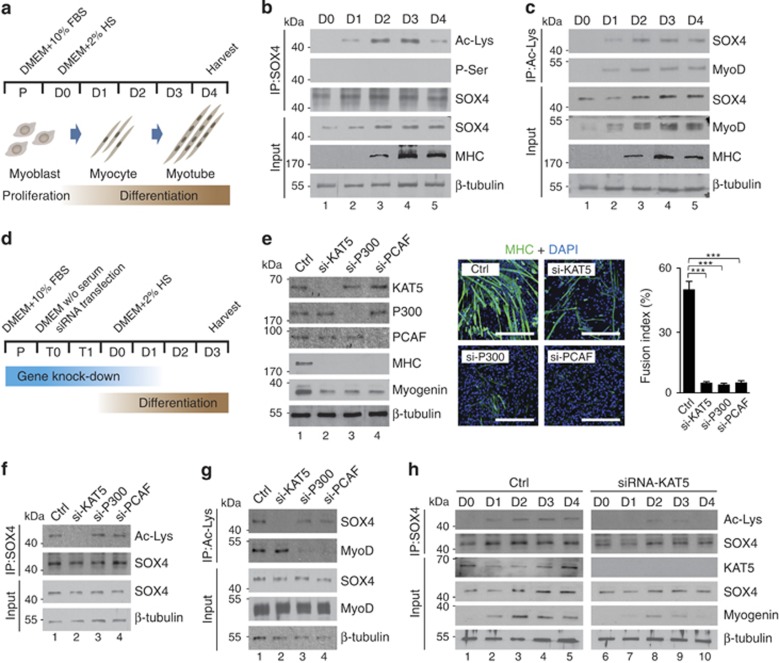
KAT5 is required for SOX4 acetylation during myoblast differentiation. (**a**) Schematic diagram of myoblast differentiation into myotubes. (**b** and **c**) SOX4 acetylation during C2C12 myoblast differentiation. C2C12 myoblast lysates prepared at the indicated times after the induction of differentiation were immunoprecipitated with SOX4 (**b**) or acetyl-lysine (Ac-Lys) (**c**) antibodies and immunoblotted with each of the antibodies to detect acetylated SOX4. Myosin heavy chain (MHC) was used as a muscle-specific marker. MyoD was used as a positive marker for acetylation. *β*-Tubulin was assessed as a loading control. (**d**) Schematic diagram of gene knockdown strategy during myoblast differentiation. (**e**) Requirement for acetyltransferase enzyme activity during myogenesis. C2C12 myoblasts were transfected with scrambled-siRNA (Ctrl), KAT5-, P300-, or PCAF-siRNA and were induced to differentiate by incubation for 3 days in differentiation medium. The efficacy of KAT5, P300, and PCAF knockdown with siRNA as well as the myoblast differentiation rates were assessed by immunoblot analysis. MHC immunostaining and quantified fusion index shows muscle cell differentiation. Scale bar indicates 400 *μ*m. ***P<0.001. (**f** and **g**) Total cell lysates from the differentiated myoblast cells described in (**e**) were immunoprecipitated with antibody against Sox4 (**f**) or acetyl-lysine (**g**) and immunoblotted with Ac-Lys or Sox4 antibodies. (**h**) Dynamic SOX4 acetylation patterns were detected by the time course of myoblast differentiation in scrambled- or KAT5-siRNA-transfected C2C12 cells

Although SOX4 was expressed in undifferentiated myoblast cells, its transcriptional activity was fully reached only after differentiation. In this regard, PTMs of SOX4 (SOX4-PTMs) may be critical for its functional activation. To test this, we differentiated C2C12 myoblast cells into myotubes by exchanging the conditional medium with 2% horse serum for 4 days (Figure 1a), then observed the SOX4-PTMs. Surprisingly, SOX4 acetylation increased 2 days after differentiation by immunoprecipitation of SOX4 (Figure 1b). However, SOX4 phosphorylation (phosphor-Ser) was not detectable (Figure 1b; Supplementary Figure 1a). The presence of acetylated-SOX4 was further confirmed by reciprocal immunoprecipitation with anti-Ac-lysine antibodies (Figure 1c; Supplementary Figure 1b).

Because HAT proteins, such as P300/CBP and PCAF, are involved in the acetylation of MyoD during myoblast differentiation,^13, 14^ we evaluated the effect of knockdown of HAT proteins on SOX4 acetylation. Undifferentiated myoblast cells were transfected with siRNAs targeting KAT5, P300/CBP, or PCAF, and then 2 days after transfection, cells were differentiated into myotubes for 3 days (Figure 1d). Expression of HAT proteins was successfully abolished, and these cells showed severe myotube differentiation defects with low myosin heavy chain staining and 90% decrease of myotube fusions (Figure 1e). The acetylated SOX4 level was markedly decreased only by the knockdown of KAT5 (Figures 1f and g). Furthermore, immunoprecipitation of SOX4 during the process of differentiation showed that KAT5 significantly regulated the acetylation of SOX4 (Figure 1h).

### Association of KAT5 and SOX4

**Figure 2:**
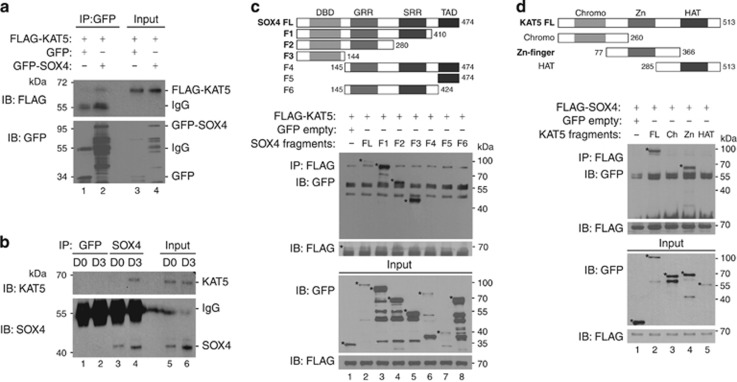
KAT5 physically interacts with SOX4. (**a**) HEK293 cells were transfected with FLAG-KAT5 along with a GFP vector or with GFP-SOX4 expression plasmids. Total cell lysates were immunoprecipitated with GFP antibodies, and co-precipitated KAT5 was detected with anti-FLAG antibodies. (**b**) Total cell lysates from proliferating myoblast (D0) or differentiating (D3) C2C12 cells were immunoprecipitated with endogenous SOX4 antibodies, and co-precipitated KAT5 was detected with immunoblot analysis using KAT5 antibodies. (**c**) The top panel shows a schematic representation of the SOX4 fragments used in this binding analysis. GRR, glycine-rich region; SRR, serine-rich region; TAD, transactivation domain. Plasmids encoding FLAG-KAT5 and GFP-fused SOX4 full-length (FL) or SOX4 fragments (F1-F6) were transfected into HEK293 cells. Total cells lysates were immunoprecipitated with anti-GFP antibodies and immunoblotted with anti-FLAG antibodies. (**d**) The top panel shows a schematic diagram of KAT5 protein. FL, full-length of KAT5; Chromo, chromodomain; Zn, zinc-finger domain. Plasmids encoding FLAG-SOX4 and each of the GFP-KAT5 truncation mutants were transfected into HEK293 cells. Total cell lysates were immunoprecipitated with anti-FLAG antibodies and immunoblotted using anti-GFP antibodies

Because KAT5 regulates the acetylation level of SOX4, we next investigated KAT5–SOX4 physical interaction. Co-immunoprecipitation showed that KAT5 precipitated with SOX4 and *vice versa* (Figure 2a; Supplementary Figure 2a). KAT5 and SOX4 interacted at the onset of differentiation (D3) but not during the proliferation stage (D0) (Figure 2b). To examine the specific binding region for the interaction between SOX4 and KAT5, HEK293 cells were transfected with GFP-fused full-length SOX4 (SOX4 FL) or various SOX4 fragments (F1~F6) together with FLAG-KAT5 (Figure 2c). Co-immunoprecipitation demonstrated that SOX4 FL and SOX4 fragments F1-F3, which contain the DNA-binding domain (DBD), are required for the association with KAT5 (Figure 2c, Supplementary Figure 2c). We also performed co-immunoprecipitations using SOX4 FL and various KAT5 fragments (Figure 2d). SOX4 precipitated with full-length KAT5 and with the KAT5 zinc finger domain, but not with the chromodomain or HAT domain. These results indicate that the DBD in SOX4 and the zinc finger domain of KAT5 are required for physical co-association between SOX4 and KAT5.

### KAT5 acetylates SOX4 at Lys95 residue

**Figure 3:**
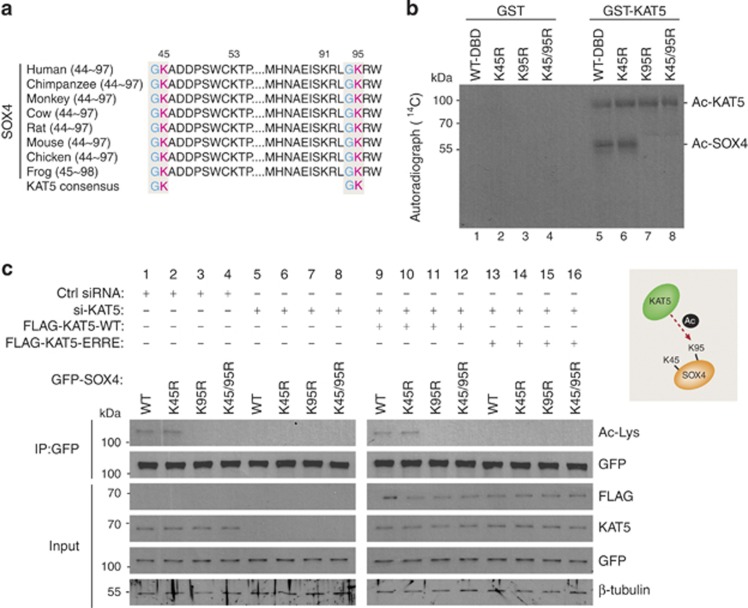
KAT5 directly acetylates Lys95 residue of SOX4 *in vitro* and *in vivo*. (**a**) Sequence comparison of SOX4 DBD among various species. KAT5 consensus amino acids and the putative Lys45 and Lys95 acetylation sites in SOX4 are indicated by shading. (**b**) Purified GST-Sox4 DBD WT or with point mutations (K45R, K95R, or K45/95R) was incubated with purified GST-KAT5 or GST only, along with ^14^C acetyl-CoA. Coomassie blue staining was used to demonstrate equal loading of proteins (Supplementary Figure 3a). (**c**) C2C12 myoblasts were transfected with indicated plasmids and maintained in the differentiation medium for 3 days. Whole-cell lysates from these transfectants were immunoprecipitated with anti-GFP antibodies and immunoblotted with anti-Ac-Lys antibodies

Because SOX4 physically interacts with KAT5, we next tested whether KAT5 acetylates SOX4. A previous study proposed that lysine residues near glycine/alanine residues are targets of KAT5-mediated acetylation.^27^ Two of the lysine residues (Lys45 and Lys95) of Sox4 are adjacent to glycine/alanine residues, and also well conserved across species (Figure 3a). Thus, we replaced Lys45 and Lys95 with arginine, and performed an *in vitro* acetylation analysis (Figure 3b). The level of acetylation of the SOX4-K45R mutant protein was not changed compared with SOX4 wild type (WT) (Figure 3b, lane 6). However, acetylation was markedly abolished in the K95R and K45/95R mutants (Figure 3b, lanes 7 and 8). KAT5 self-acetylation was also detected (Figure 3b, lanes 5–8). Although P300 interacts with SOX4,^33^ acetylation of SOX4 was hardly detected by P300 HAT activity (Supplementary Figure 2b). These results demonstrate that Lys95 in SOX4 is a direct target of KAT5-mediated acetylation.

To further confirm that Lys95 of SOX4 is acetylated by KAT5 during myoblast differentiation, C2C12 cells were transfected with scrambled-siRNA (ctrl-siRNA) or KAT5-siRNA along with WT-SOX4 or SOX4 lysine mutants (Figure 3c). Consistent with the *in vitro* analysis, the Lys95 and Lys45/95 SOX4 mutants were not acetylated in the ctrl-siRNA-transfected cells (Figure 3c, lanes 3 and 4), whereas the Lys45 mutation did not affect SOX4 acetylation (Figure 3c, lane 2). However, the knockdown of endogenous KAT5 significantly abrogated the acetylation of all of the tested SOX4 proteins including SOX4-WT and the Lys45 mutant (Figure 3c, lanes 5–8). We also tested KAT5-specific acetylation of SOX4 using a KAT5 acetyltransferase mutant (KAT5-ERRE) (Figure 3c). To avoid endogenous effects of KAT5, C2C12 cells were transfected with KAT5 siRNA, and then KAT5-WT or KAT5-ERRE mutant-expressing plasmids were overexpressed. Only KAT5-WT could acetylate the Lys95 residue of SOX4 (Figure 3c; lanes 9 and 10), but not with the KAT5-ERRE (Figure 3c, lanes 13–16; Supplementary Figure 3b).

### KAT5-mediated acetylation of SOX4 at Lys95 is critical for myoblast differentiation

**Figure 4:**
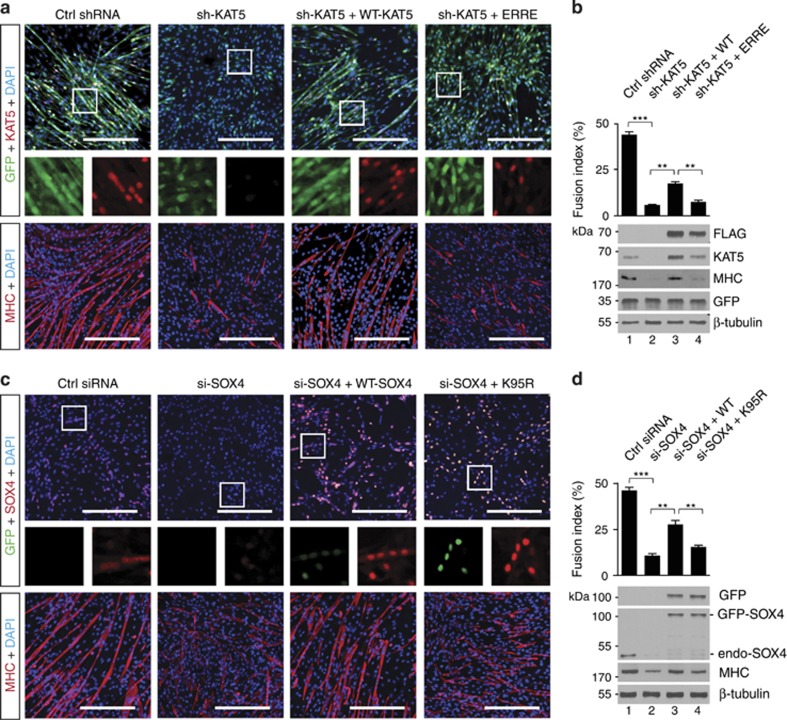
Acetylation of SOX4 at Lys95 is critical for myoblast differentiation. (**a**) C2C12 myoblast cells were transfected with control shRNA (Ctrl shRNA) or KAT5 shRNA (sh-KAT5) along with FLAG-KAT5 (WT or ERRE HAT mutants) to rescue KAT5 expression and maintained for 3 days under differentiation conditions. GFP expression shows transfection efficiency as the GFP coding region is involved in shRNA vector constructs. KAT5 and myosin heavy chain (MHC) expression was detected by immunocytochemical analysis. Scale bar indicates 400 *μ*m. (**b**) Fusion index and immunoblot analysis of the cell lysates as described in (**a**). **P<0.01; ***P<0.001. (**c**) Control siRNA (Ctrl-siRNA) or SOX4-siRNA along with GFP-SOX4 (WT or K95R) to rescue SOX4 expression, maintained for 3 days under differentiation conditions. SOX4 and MHC expression was assessed with immunofluorescence analysis. Scale bar indicates 400 *μ*m. (**d**) Fusion index and immunoblot analysis of the cell lysates as described in (**c**). **P<0.01; ***P<0.001

We next investigated whether KAT5-mediated SOX4 acetylation is required for myoblast differentiation. To check the transfection efficiency and cellular morphology simultaneously, C2C12 cells were transfected with GFP-KAT5-shRNA (Figure 4a). In contrast to the control group (Figure 4a; Supplementary Figure 4a), morphological myotube alignment was not observed in the KAT5 shRNA-transfected cells (Figure 4a). Myosin heavy chain staining showed that KAT5-shRNA-transfected myoblast cells were not fully differentiated (Figure 4a). To test the importance of KAT5 enzyme activity, we reconstituted KAT5 expression with WT-KAT5 or KAT5-ERRE under the KAT5 knockdown background (Supplementary Figure 4b). WT-KAT5 rescued myotube formation morphologically, and the fusion index was increased by about 40% compared with the KAT5-shRNA-transfected group (Figures 4a and b). In contrast, HAT activity-deficient KAT5 did not increase the fusion index, and cellular morphology showed undifferentiated shape similar to that in the KAT5-shRNA transfected group (Figures 4a and b). Moreover, we tested the effect of SOX4 acetylation at Lys95 during myoblast differentiation. Knockdown of SOX4 significantly reduced myotube formation (Figure 4c; Supplementary Figure 4c). Myotube formation was rescued to around 50% by WT-SOX4 transfection under the SOX4 knockdown background (Figures 4c and d; Supplementary Figure 4d). However, the transfection with SOX4 mutation at Lys95 did not rescue myotube formation from the SOX4 knockdown condition (Figures 4c and d; Supplementary Figure 4d).

### KAT5-mediated SOX4 acetylation is required for full transcriptional activity of SOX4 during myoblast differentiation

**Figure 5:**
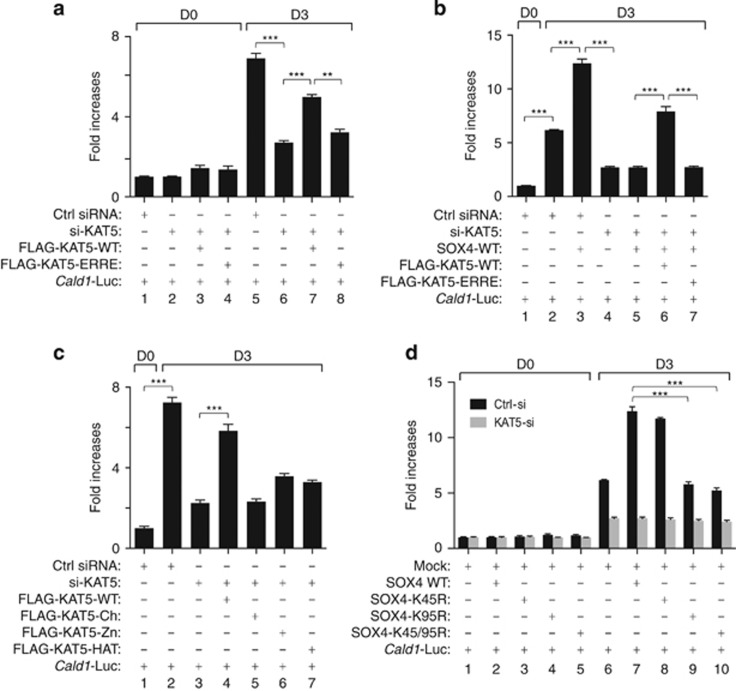
KAT5-mediated SOX4 acetylation regulates the transcriptional activity of SOX4 during myogenesis. (**a–d**) The *Cald1* promoter-containing luciferase vector and the indicated constructs were transfected into C2C12 myoblast cells, and the transfectants were maintained under differentiation conditions for 3 days. Promoter activities are expressed as fold changes compared with activation in control siRNA-transfected cells under undifferentiating condition. **P<0.01; ***P<0.001

As *Cald1* is a downstream target of SOX4,^7^ we used the *Cald1* promoter to examine the effect of SOX4 acetylation. In the differentiated state, *Cald1* promoter activity was increased sevenfold owing to endogenous SOX4 and KAT5 (Figure 5a, lane 5) compared with that in undifferentiated cells (Figure 5a, lane 1). However, *Cald1* promoter activity was significantly reduced by the knockdown of endogenous KAT5 (Figure 5a, lane 6). Overexpression of WT-KAT5 reconstituted the promoter activity of *Cald1*, but the KAT5 mutant did not (Figure 5a, lanes 7 and 8). To test SOX4-specific transcriptional activity on the *Cald1* promoter, we overexpressed WT SOX4 in differentiated C2C12 cells (Figure 5b). Promoter activity of *Cald1* was increased around 12-fold (Figure 5b, lane 3) compared with the undifferentiated group (Figure 5b, lane 1). Knockdown of KAT5 significantly reduced the promoter activity of *Cald1* (Figure 5b, lane 5). Reduced promoter activity was recovered by the transfection of WT-KAT5 but not with the ERRE mutant (Figure 5b, lanes 6 and 7). To further test the importance of the physical interaction between KAT5 and SOX4, C2C12 cells were transfected with various KAT5 fragments (Figure 5c). To avoid the endogenous effects of KAT5, we tested the promoter activity under the KAT5 knockdown background. Only full-length KAT5 rescued around 80% of the transcriptional activity of SOX4 that was decreased by KAT5 knockdown (Figure 5c, lane 4). Furthermore, we next tested whether SOX4 acetylation at Lys95 residue is critical for SOX4 activity (Figure 5d). SOX4 mutants that include the K95R point mutation did not restore SOX4 transcriptional activity (Figure 5d, lanes 9 and 10). In contrast, SOX4 mutants that include the K45R mutation did restore the transcriptional activity of SOX4, which demonstrates the importance of SOX4 acetylation at Lys95 residue (Figure 5d, lane 8).

### Chromatin remodeling by KAT5 is required for SOX4-mediated *Cald1* promoter activation during C2C12 myoblast differentiation

**Figure 6:**
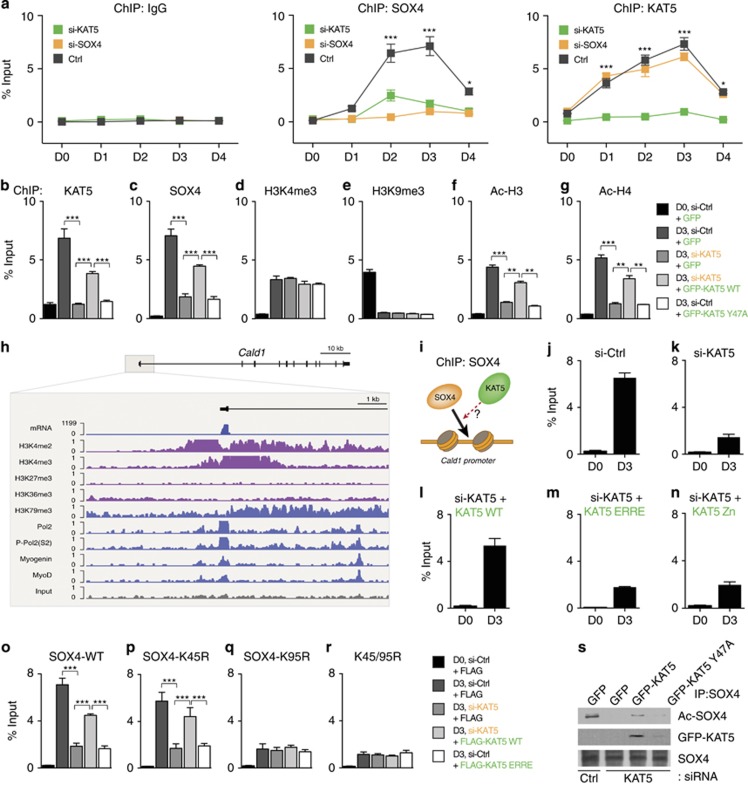
KAT5 triggers myoblast differentiation through chromatin remodeling and SOX4 acetylation. (**a**) Recruitment of SOX4 and KAT5 to *Cald1* promoter during myoblast differentiation. C2C12 myoblast cells were transfected with scrambled siRNA (Ctrl), KAT5 (si-KAT5), or SOX4-siRNA (si-SOX4) and incubated under differentiation conditions for the indicated times (differentiation days D0 to D4). SOX4 and KAT5 occupancy at the *Cald1* promoter was analyzed with ChIP assays using anti-SOX4 and KAT5 antibodies. (**b–g**) Chromodomain-dependent recruitment of KAT5 to the *Cald1* promoter during myoblast differentiation was assessed by ChIP analysis using indicated specific antibodies. C2C12 myoblast cells were transfected with KAT5-siRNA with or without GFP-KAT5 WT or the chromodomain mutant (Y47A) to restore KAT5 expression. After incubation in D0 or D3 conditions, ChIP analysis was performed to examine the recruitment of KAT5 to the *Cald1* promoter as indicated. (**h**) The chromatin environment of the *Cald1* promoter was further confirmed from the mouse ENCODE project. ChIP-seq signals were visualized on the UCSC genome browser and modified. (**i–n**) SOX4 recruitment to the *Cald1* promoter was mediated by KAT5 HAT enzyme activity-dependent chromatin opening. C2C12 myoblast cells were transfected and differentiated as indicated in the figure, and ChIP analysis was performed using SOX4 antibodies. (**o–r**) GFP-SOX4 WT or its point mutants were transfected into C2C12 myoblasts, and ChIP analysis was performed using GFP antibodies after differentiation as indicated times. (**s**) GFP-KAT5 WT or Y47A mutants were transfected into KAT5-silenced C2C12 myoblast cells and maintained in the differentiation medium for 3 days. Cell lysates were immunoprecipitated with SOX4 antibodies, and bound KAT5 or acetylated SOX4 was detected by immunoblot analysis using KAT5 or Ac-Lys antibodies. *P<0.05; **P<0.01; ***P<0.001

In addition to acetylating SOX4, we tested whether KAT5 modifies the chromatin structure thereby increasing TF access to the *Cald1* promoter. As shown in Figure 6a, the recruitment of KAT5 and SOX4 to the *Cald1* promoter occurred as soon as the cells were placed in differentiation medium (D1) and increased as the myoblast differentiated (D3). In contrast, KAT5-silenced cells failed to recruit SOX4 to the *Cald1* promoter (Figure 6a, middle, green), suggesting that KAT5 is required to allow SOX4 to access the *Cald1* promoter during myoblast differentiation.

We next examined whether the recognition of methylated histones by the chromodomain of KAT5 is critical for chromatin opening and SOX4 recruitment. To test this, C2C12 cells were transfected with KAT5-WT or a KAT5 chromodomain mutant (Y47A) under the KAT5 knockdown background, and then molecular occupancies at the *Cald1* promoter region were analyzed by chromatin immunoprecipitation (ChIP) using indicated antibodies. KAT5 and SOX4 recruitment on the promoter region was significantly reduced by the knockdown of KAT5 (Figures 6b and c, lane 3), but their molecular associations were rescued around 50% by the expression of KAT5-WT (lane 4). In contrast, the chromodomain mutant of KAT5 (Y47A) did not rescue the molecular recruitment of KAT5 and SOX4 (lane 5). Both active and repressive histone markers, H3K4me3 and H3K9me3, respectively, were not affected by the knockdown of KAT5 (Figures 6d and e). Through myoblast differentiation, the active histone marker H3K4me3 was significantly increased instead of reduction of the repressive histone marker H3K9me3 (Figures 6d and e). In contrast to histone methylation, histone acetylation of H3 and H4 were significantly affected by KAT5 knockdown and chromodomain mutant expression (Figures 6f and g, lanes 3 and 5). The chromatin environment and mRNA expression on the *Cald1* promoter was further confirmed by a previous genome-wide analysis.^34^ ChIP-seq results with modified histones, Pol II, MyoD, and Myogenin in differentiated C2C12 cells were visualized on the UCSC genome browser (Figure 6h). Protein recruitment patterns are well matched with our ChIP-qPCR results on the *Cald1* promoter region (Figure 6h).

As we revealed that the presence of KAT5 is critical for SOX4 recruitment on the *Cald1* promoter region (Figure 6a), we next tested the molecular mechanisms underlying KAT5-mediated SOX4 recruitment (Figure 6i). SOX4 recruitment to the promoter area was significantly reduced in KAT5 knockdown cells (Figures 6j and k); however, SOX4 binding affinity recovered by 80% after KAT5-WT reconstitution (Figure 6l). Both the KAT5-HAT activity mutant and the Zn-fragment of KAT5, which has the SOX4 binding domain without the HAT domain, could not recover SOX4 recruitment (Figures 6m and n). Furthermore, SOX4 loading on the *Cald1* promoter was highly depended on its acetylation status by KAT5. C2C12 cells were transfected with GFP-SOX4-WT or GFP-SOX4 mutants together with KAT5-WT or HAT mutants, and ChIP was performed using anti-GFP antibodies. SOX4 recruitment on the *Cald1* promoter was increased in differentiated myoblast cells (Figure 6o, lane 2), and its loading patterns were dependent on KAT5 enzyme activity (Figure 6o, lanes 3–5). The SOX4-K45R mutant-overexpressed groups showed similar SOX4 recruitment patterns (Figure 6p) as that with SOX4-WT groups (Figure 6o). However, the SOX4-K95R and K45/K95R mutant groups showed significantly reduced SOX4 recruitment on the *Cald1* promoter region (Figures 6q and r). Moreover, KAT5-Y47A mutant showed deficient differentiation rates (Supplementary Figure 5) together with decreased acetylation rates of SOX4 and SOX4 interaction compared with KAT5-WT (Figure 6s, lanes 3 and 4). These results suggest that chromatin remodeling mediated by KAT5 is required for SOX4–KAT5 interaction and KAT5-mediated SOX4 acetylation, resulting in SOX4-dependent *Cald1* promoter activation during myoblast differentiation.

### SOX4-centered molecular switching between KAT5 and HDAC1 during myoblast differentiation

**Figure 7:**
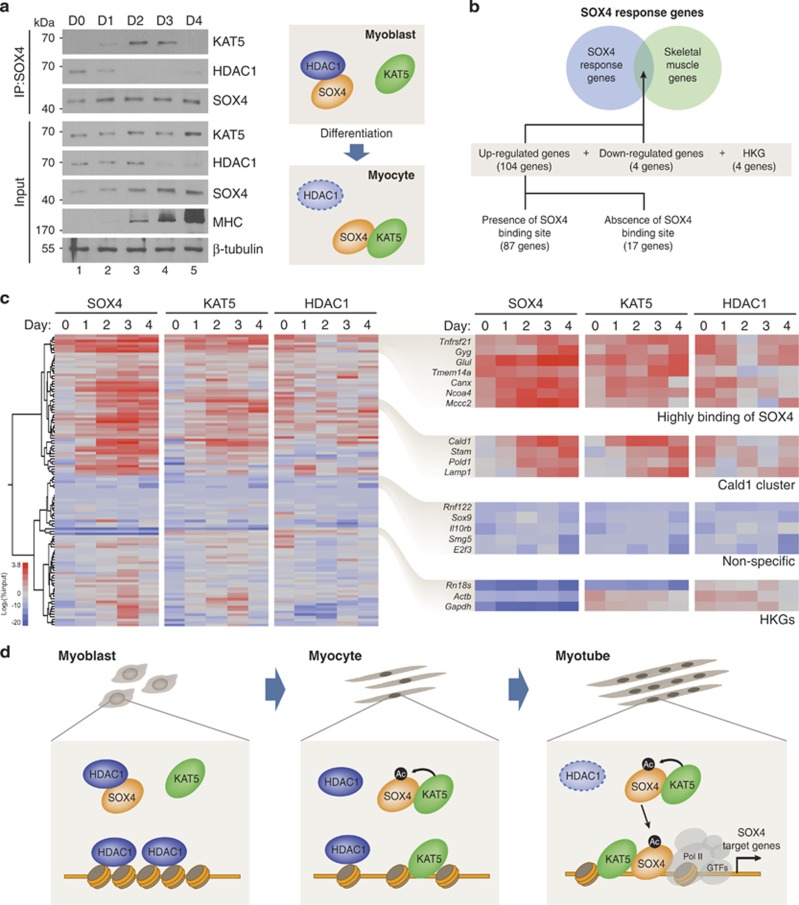
SOX4 function is regulated by molecular switching between KAT5 and HDAC1 during myoblast differentiation. (**a**) Differentiated C2C12 cells were immunoprecipitated with SOX4 antibodies and co-precipitated KAT5 or HDAC1 was detected by immunoblot analysis using its specific antibodies. (**b**) Schematic diagram of procedure of SOX4 response gene selection for ChIP analysis. Genes which have no SOX4-binding sites, and housekeeping genes (HKG) were used as a negative control. (**c**) Systematic ChIP analysis using anti-SOX4, KAT5, and anti-HDAC1 antibodies. The ChIP results for each promoter are presented in a heat map and hierarchical clustering dendrogram for D0 to D4 during C2C12 myoblast differentiation. The heat map shows log_2_(% input) after ChIP-qPCR. Genes with *P*-values of ≤0.1 and a mean of normalized percentage compared with input signal were selected. Blue indicates low binding and red indicates high binding on each gene promoters. (**d**) Proposed model for SOX4 transactivation by the molecular switching between KAT5 and HDAC1 during myoblast differentiation. SOX4 interacts with HDAC1 in proliferating C2C12 myoblasts (left panel). In response to myoblast differentiation signals, HDAC1 is dissociated from SOX4, which provides chance for acetylation via interaction with KAT5. In same times, histones acquire active marks through the combined actions of methyltransferase and demethylase, and KAT5 is recruited via recognition of H3K4me3 by its chromodomain. KAT5 then acetylates histone H3 and H4, which leads to the opening of compacted histone-DNA structures to provide the SOX4 binding site platform (middle panel). Finally, acetylated SOX4 is recruited at the target gene promoter, leading to full activation of the transcription machinery (right panel)

The observation that SOX4 acetylation levels are lower in proliferating C2C12 cells than in myotubes suggest a role for HDACs in modulating SOX4 acetylation. Although acetylated SOX4 was not recovered by TSA treatment (Supplementary Figure 6a), we found that HDAC1 specifically interacts with SOX4 in myoblast cells, and this interaction was decreased during C2C12 differentiation (Supplementary Figure 6b). Without decreasing the SOX4 expression level (Supplementary Figure 6c), HDAC1 overexpression leads to the decrease of KAT5-mediated SOX4 transcriptional activity in HEK293 cells (Supplementary Figure 6d), suggesting that HDAC1 may block the KAT5–SOX4 interaction via competitive mechanisms to occupy the SOX4 DBD region (Supplementary Figure 6e). However, the HDAC1–SOX4 or KAT5–SOX4 interaction was not affected by KAT5 or HDAC1 overexpression (Supplementary Figures 6f and h). To test molecular switching instead of competition, total cell lysates of differentiated C2C12 were immunoprecipitated with anti-SOX4 antibodies at indicated time points, and protein interactions were visualized. HDAC1 to SOX4 interaction was reduced, and SOX4 became a co-activator for KAT5 during C2C12 differentiation (Figure 7a).

To elucidate protein recruitment patterns involving SOX4, KAT5, and HDAC1 during myoblast differentiation, we selected 112 putative SOX4 target genes that contain SOX4-binding sequences in their promoter regions (within 1 kb upstream of the transcription start site) (Figure 7b). The selected genes were categorized into eight subgroups based on the Gene Ontology annotation (Supplementary Figure 7a). Interestingly, 32% of the genes were found to function in cell signaling/migration/adhesion pathways that are involved in myotube formation.^35^ ChIP was then performed using anti-SOX4, HDAC1, and KAT5 antibodies at the indicated differentiation time points (Figure 7c). Hierarchical clustering analysis of the ChIP results indicated that putative SOX4 target genes largely separated into three groups: early, late, and no response. In the early and late response groups, SOX4 and KAT5 bound concurrently to each of the SOX4 target genes (Figure 7c). *Stam*, *Pold1*, and *Lamp1* clustered with *Cald1*, a known SOX4 target gene,^7^ as did genes with similar association patterns in the early response group (Figure 7c). SOX4 and KAT5 showed high occupancy at the promoters of *Tnfrsf21*, *Gyg*, *Glul*, and *Tmem14a* genes (Figure 7c). Cell signaling, migration, and adhesion-related genes are 17 (31.5%) out of the early response genes (48.2% of the selected) and 14 (40%) out of the late response genes (31.3% of the selected) (Supplementary Figure 7b). The gene ontology categories were more evenly distributed in the no response gene group (Supplementary Figure 7b). Taken together, concurrent binding of SOX4 and KAT5 to SOX4 target gene promoters was specifically enriched at genes related to cell signaling, migration, and adhesion during myoblast differentiation.

## Discussion

The timely and accurate expression of subsets of genes by intrinsic factors drives skeletal muscle differentiation in response to extrinsic cues. PTMs of intrinsic and extrinsic factors control their regulatory signals from ‘on/off switching' to ‘gradual switching', and it increases the variety in biological reactions. Among various PTMs, switching of molecular signaling by acetylation is well studied with HAT enzymes such as P300/CBP, PCAF, and MYST family members.^8, 9, 36^ HATs also acetylate non-histone proteins including TFs.

In the proliferating myoblast cells, MyoD transcriptional activity is inhibited by methylation at Lys104 residue mediated by G9a methyltransferase.^37^ In contrast, increased MyoD acetylation at several lysine residues correlates with transcriptional activity during differentiation.^13, 14^ We demonstrate that SOX4 acetylation is also critically associated with myoblast differentiation. During C2C12 differentiation, KAT5 specifically acetylates SOX4, which is gradually increased during the differentiation (Figure 1). Conversely, knock-down of KAT5 decreases SOX4 acetylation, and inhibits myoblast differentiation (Figures 3 and 4). Previous reports showed that KAT5 is a critical co-activator in developmental pathways (Hattori *et al.*,^39^) and modulated the transcriptional activities of PAX6 and neural leucine zipper, which are key factors for retinal development.^24, 25, 38^ In mouse embryonic development, KAT5 is also expressed at moderate levels in various organs including skeletal muscle.^40^ In this regard, understanding of SOX4 acetylation by KAT5 will provide insight into how SOX4 is temporally regulated under differentiation conditions.

Although the acetylation of several TFs such as p53 and MyoD has been connected to their DNA-binding activity at target gene promoters,^41^ the acetylation of SOX family proteins has been reported to affect cellular and nuclear localization rather than controlling of binding affinity to target gene promoters.^42^ These reports suggest that acetylation sites of SOX proteins within an NLS or NES are important for their localization but not for binding to target genes. We confirmed in this study that SOX Lys95 is located in the NES^42^ by SOX4 amino acid sequence alignment, suggesting that SOX4 acetylation may regulate cellular localization. However, our results also showed that SOX4 is partially localized in the cytosol, and enriched in the nucleus regardless of its acetylation status (Figure 4c). Because the hydrophobic residues of SOX4, which are recognized by nuclear export proteins, are absent from this NES,^42, 43^ SOX4-NES may not be functional. Moreover, SOX4-Lys95 is conserved in several SOX proteins including SOX7, SOX8, SOX9, SOX10, and SOX11; thus, the physiological role of SOX4 acetylation is dependent on ‘which residue is modified'. Our results indicate that SOX4-Lys95 is supposed to be critical for binding to target genes and transcriptional activity.

KAT5 has been reported to function as a HAT and as a chromatin reader via its chromodomain.^18^ The interaction of chromodomain-containing proteins with methylated histone residues facilitates the recognition and interpretation of the chromatin histone code.^35, 44, 45, 46^ The direct interaction between the chromodomain of KAT5 and H3K9me3 at DNA double-strand breaks increases KAT5 acetyltransferase activity.^47^ Moreover, our previous studies showed that KAT5 interacts with target gene promoters independently on TF recruitment,^25, 26, 36^ suggesting that KAT5 can immediately remodel the chromatin structure via its chromodomain to facilitate the recruitment of TFs to their target promoters. Our results in this study indicate that WT KAT5 recognizes H3K4me3 within the *Cald1* promoter, leading to histone H3 and H4 acetylation and chromatin opening (Figure 6). Thus, KAT5 chromodomain-dependent recognition of H3K4me3 can serve as a key bridge between the epigenetic histone code and TFs. Identification of the histone methyltransferase or demethylase that establishes the H3K4me3 platform for KAT5 recognition in response to myoblast differentiation signals may help to elucidate the full mechanism of transcriptional regulation – from histone configuration to TF activity.

Taken together, our results suggest the following sequence of events during the early stages of SOX4 transactivation (Figure 7d): (i) SOX4 interacts with HDAC1 in proliferating myoblasts; (ii) In response to differentiation signal, HDAC1 dissociates from SOX4, and free SOX4 is acetylated via interaction with KAT5. In addition, KAT5 recognizes and binds to H3K4me3, a histone mark created by other histone-modifying enzymes, within the *Cald1* promoter through its chromodomain. Histone acetylation mediated by KAT5 HAT activity opens compacted histone-DNA structures; (iii) Acetylated SOX4 is recruited to the *Cald1* promoter, leading to full activation of the transcription machinery. Our results elucidate how the multiple functions of KAT5 in chromatin remodeling, histone modification, protein–protein interaction, and interaction-dependent acetylation are coordinated to promote the formation of an active SOX4 transcriptosome at target gene promoters during myoblast differentiation.

## Materials and Methods

### Cell culture and plasmid transfection

Human embryonic kidney 293 (HEK293) cells and C2C12 cells, a myogenic cell line derived from mice, were obtained from the American Type Culture Collection (Manassas, VA, USA) and maintained in Dulbecco's modified Eagle's medium supplemented with 10% fetal bovine serum and penicillin-streptomycin (50 units/ml; Invitrogen, Carlsbad, CA, USA) in a humidified atmosphere of 5% CO_2_ at 37 °C. C2C12 myoblast differentiation was induced in differentiation medium (Dulbecco's modified Eagle's medium supplemented with 2% horse serum). Freshly trypsinized cells were transiently transfected with a 2:1 ratio (*μ*l : *μ*g) of Lipofectamine 2000 (Invitrogen) to plasmid DNA immediately after plating.^48^

### Constructs and antibodies

The mouse *Cald1* promoter was cloned into the pGL.4.12 luciferase vector as described previously.^7^ The coding regions of WT and mutant KAT5, including the truncation, ERRE, and Y47A mutants, were cloned into a pFLAG-CMV2 (E7033; Sigma-Aldrich, St. Louis, MO, USA) or pEGFPC2 (6083; Clontech, Mountain View, CA, USA) vector. cDNA encoding WT SOX4, SOX4 truncation mutants, and SOX4 point mutants (including K45R, K95R, and K45/95R) was introduced into a pFLAG-CMV2 or pEGFPC2 vector. SOX4 siRNA was cloned into the pBabe-dual vector as described previously.^7^ P300/CBP and PCAF siRNA were purchased from Santa Cruz Biotechnology (Santa Cruz, CA, USA). KAT5 siRNA and shRNA targeting the 3′-untranslated region of mouse *Kat5* mRNA were introduced into the pBabe-dual vector or psiRNA-hH1GFPzeo G2 vector (Invivogen, San Diego, CA, USA). The following antibodies were purchased from Santa Cruz Biotechnology: anti-MyoD, anti-FLAG, anti-phospho-serine, anti-acetyl-lysine, anti-glutathione S-transferase (GST), anti-P300/CBP, anti-PCAF, and anti-*β*-tubulin. The following antibodies were purchased from Millipore (Temecula, CA, USA): anti-acetyl histone H3 and H4 and anti-trimethyl-histone H3K4, H3K9, and H3K27. The following commercially available antibodies were used: anti-myosin heavy chain (Developmental Studies Hybridoma Bank, Iowa City, IA, USA), anti-KAT5 and anti-FLAG (Sigma, St. Louis, MO, USA), and anti-GFP (Roche, Mannhein, Germany). Anti-SOX4 was generated using a SOX4-specific peptide as described previously.^7^

### Luciferase assay

C2C12 cells were transfected with vectors containing a firefly luciferase reporter gene (0.1 *μ*g) and pCMV-*β*-galactosidase (0.1 *μ*g) along with different plasmids using Lipofectamine 2000 (Invitrogen). Transfected cells were lysed in reporter lysis buffer (Promega, Madison, WI, USA), and cell extracts were analyzed with a luciferase reporter assay system using a GloMax luminometer (Promega). Luciferase activities were normalized to the *β*-galactosidase activity of the co-transfected vector.

### Immunoprecipitation and immunoblotting

The cells were lysed in a buffer containing 1% Triton X-100, 150 mM NaCl, 50 mM Tris-HCl, pH 7.5, 0.1% sodium dodecyl sulfate (SDS), 1% Nonidet P-40, and 1 mM PMSF. The cell suspensions were incubated on ice for 20 min and centrifuged at 12 000 r.p.m. at 4 °C for 20 min. For immunoprecipitation assays, the supernatants were precleaned with 20 *μ*l of protein A/G-agarose bead (50% slurry) and then incubated at 4 °C overnight with 40 *μ*l of fresh protein A/G bead in the presence of appropriate antibodies. The beads were washed three times in PBS, resuspended in SDS sample buffer, and boiled for 10 min. The protein samples were electrophoresed on a 10% SDS-polyacrylamide gel and were transferred to a nitrocellulose membrane (PROTRAN, Whatman, Pittsburg, PA, USA). The membrane was blocked with 5% skim milk in a solution of 20 mM Tris-HCl, pH 7.6, 137 mM NaCl, and 0.1% Tween 20 and incubated with appropriate dilutions of the primary antibody at room temperature for 3 h. Samples were analyzed by western blotting using the appropriate antibodies to detect protein expression. The original blotting results are shown in Supplementary Figure 8.

### Immunofluorescence assay

Immunofluorescence assay was performed following a protocol provided by Abcam (Cambridge, MA, USA). C2C12 cells were grown on a sterile coverslip in 60-mm dishes and transfected with indicated plasmid vectors using Lipofectamine 2000. Differentiating C2C12 cells in differentiation medium were fixed with 4% paraformaldehyde and incubated with appropriate antibodies (1 : 200) followed by Cy3-conjugated anti-mouse and anti-rabbit secondary antibodies (Jackson ImmunoResearch Laboratories, West Grove, PA, USA). Plates were washed three times in PBS, and confocal imaging was performed with a Zeiss (Salt Lake City, UT, USA) LSM 700 confocal microscope.

### Protein purification and *in vitro* acetylation assay

All recombinant proteins (GST, GST-SOX4 WT DBD, GST-SOX4 DBD point mutants, and GST-KAT5) were purified from *Escherichia coli*. For the *in vitro* acetylation assays, the reactions were assembled in lysine acetyltransferase buffer with purified proteins and ^14^C-acetyl-CoA (55 mci/mmol; Amersham, Pittsburg, PA, USA). The reaction mixtures were incubated for 1 h at 30 °C, boiled, and separated with SDS-PAGE. Proteins were visualized with autoradiography.

### Chromatin immunoprecipitation assay

The ChIP assays were conducted according to the protocol provided by Millipore. Briefly, proliferating or differentiating C2C12 cells were cross-linked with 1% paraformaldehyde (#15710, Electron Microscopy Sciences, Hatfield, PA, USA) in PBS for 15 min at 37 °C. The cells were then washed with ice-cold PBS and resuspended in 200 *μ*l of SDS-sample buffer containing a protease inhibitor mixture. The suspension was sonicated three times for 10 s with a 1-min cooling period on ice to shear chromatin to 500–1000 bp fragments, after which it was precleared with 20 *μ*l of protein A/G-agarose beads blocked with sonicated salmon sperm DNA for 30 min at 4 °C. The beads were then removed, after which the chromatin solution of each experimental group was immunoprecipitated overnight with indicated antibodies at 4 °C followed by incubation with 50 *μ*l of protein A-agarose beads (Millipore) for an additional hour at 4 °C. The immune complexes were eluted with 100 *μ*l of elution buffer (1% SDS and 0.1 M NaHCO_3_), and formaldehyde cross-links were reversed by heating at 65 °C for 4 h. Proteinase K (P2308, Sigma) was added to the reaction mixtures and incubated at 45 °C for 1 h. Immunoprecipitated and control input DNA were purified using the PCR purification kit (Qiagen, Valencia, CA, USA) and then analyzed by quantitative real-time PCR. The ChIP-PCR results were included in Supplementary Table 1, and the PCR primers used in ChIP assay and their sequences are listed in Supplementary Table 2. PCR was conducted in duplicate for each experimental condition tested.

### Statistical analysis

The data were expressed as the means±S.D. of three or more independent experiments. Statistically significant effects (*P<*0.05) were evaluated with Microsoft EXCEL software. Differences between groups were evaluated via one-way analysis of variance, followed by Student's *t*-tests, as appropriate.

## Abbreviations

KAT5: K (Lysine) acetyltransferase 5
SOX4: SRY (sex determining region Y)-box 4
HDAC1: histone deacetylase 1
MyoD: myogenic differentiation
TFs: transcription factors
IP: immunoprecipitation
ChIP: chromatin immunoprecipitation
