# Supplementary material for: KAT5-mediated SOX4 acetylation orchestrates chromatin remodeling during myoblast differentiation
Source: Cell Death Dis. 2015 Aug 20;6(8):e1857–. doi: 10.1038/cddis.2015.190 (PMC4558493; doi:10.1038/cddis.2015.190)
Supplement: Supplementary Information [file cddis2015190x1.docx]

**Supplementary information**

**Supplementary Figure 1; related to Figure 1.** (**a,b**) Differentiated C2C12 cells were immunoprecipitated with IgG and acetylated SOX4 was detected using immunoblot analysis by Ac-Lys or SOX4 antibodies.

**Supplementary Figure 2. KAT5 interacts with SOX4; related to Figure 2.** (**a**) HEK293 cells were transfected with GFP-SOX4 along with a FLAG vector or with FLAG-KAT5 expression plasmids. Total cell lysates were immunoprecipitated with FLAG antibodies, and co-precipitated SOX4 was detected with anti-GFP antibodies. (**b**) Total cell lysates from proliferating myoblast (D0) or differentiating (D3) C2C12 cells were immunoprecipitated with SOX4 antibodies, and co-precipitated P300, PCAF, and KAT5 was detected with immunoblot analysis using their specific antibodies. (**c**) FLAG-KAT5 and GFP-fused SOX4 FL or fragments were transfected into HEK293 cells. Total cell lysates were immunoprecipitated with FLAG antibodies and immunoblotted with GFP antibodies.

**Supplementary Figure 3; related to Figure 3.** (**a**) Coomassie blue staining was used to demonstrate equal loading of proteins in *in vitro* acetylation assay. (**b**) HEK293 cells were transfected with GFP-SOX4 together with FLAG-KAT5 WT or KAT5 ERRE mutants. Total cell lysates were immunoprecipitated with GFP antibodies and acetylated SOX4 was detected with immunoblot analysis using Ac-Lys antibodies. (**c**) Purified GST-SOX4 DBD was incubated with purified GST, GST-KAT5 or GST-P300 HAT domain, along with 14C acetyl-CoA. Acetylated SOX4 was expressed in phosphor image (right panel) together with Coomassie blue staining (left panel).

**Supplementary Figure 4; related to Figure 4.** (**a,c**) Knockdown efficiency of sh-KAT5 or si-SOX4, respectively. **(b,d**) KAT5- or SOX4-expressing cells to show the rescued KAT5- or SOX4.

**Supplementary Figure 5. KAT5 chromodomain is required to myoblast differentiation; related to Figure 6.** (**a**) C2C12 myoblast cells were transfected with control siRNA (Ctrl siRNA) or KAT5 siRNA (si-KAT5) along with GFP-KAT5 (wild type or Y47A chromodomain mutants) to rescue KAT5 expression and maintained for 3 days under differentiation conditions. KAT5 and MHC expression was detected by immunocytochemical analysis. (**b**) KAT5-expressing cells to show the rescued KAT5 as described in (a). (**c**) Fusion index as described in (a). (**d**) Immunoblot analysis to examine the rescued GFP-KAT5 or MHC expression as described in (a).

**Supplementary Figure 6. SOX4 is regulated by molecular switching from HDAC1 to KAT5 in response to differentiation signal; related to Figure 7.** (**a**) SOX4 acetylation is not occurred in proliferating stages. Total cell lysates from TSA-treated C2C12 myoblasts were immunoprecipitated with SOX4 antibodies and acetylated SOX4 was detected by immunoblot analysis using Ac-Lys antibodies. (**b**) HDAC1 specifically interacts with SOX4 in proliferating myoblasts. Total cell lysates from proliferating (D0) or differentiating (D3) C2C12 cells were immunoprecipitated with SOX4 antibodies and co-precipitated HDAC1-3 was detected by immunoblot analysis using their specific antibodies. (**c**) SOX4 expression was not affected by HDAC1 expression. HEK293 cells were transfected with indicated plasmid constructs and SOX4 protein rates were conducted by immunoblot analysis. GFP vector was used for transfection efficiency control. (**d**) HDAC1 reduces KAT5-mediated SOX4 transcriptional activation. HEK293 cells were transfected with indicated plasmid DNA together with *Cald1* promoter constructs, and luciferase assay was conducted. Promoter activities were expressed as fold changes compared to activation in no-transfected cells (left panel). Immunoblot analysis (right panel). (**e**) HDAC1 interacts with DBD and TAD of SOX4. Total cell lysates from transfected HEK293 cells as indicated in figure were immunoprecipitated with GFP antibodies, and co-precipitated HDAC1 was detected with Western blotting using FLAG antibodies. (**f-h**) HDAC1 and KAT5 interaction to SOX4 is not competitive binding.

**Supplementary Figure 7. GO analysis ; related to Figure 7.** (**a**) GO analysis from selected 112 genes in Figure 7B. (**b**) GO analysis of 112 selected genes by SOX4 ChIP result in Figure 7c.

**Supplementary Figure 8. Original figure files used in Fig. 1**

**Supplementary Figure 9. Original figure files used in Figs. 2 and 3**

**Supplementary Figure 10. Original figure files used in Figs. 4 and 7**

**Supplementary Table 1. ChIP results for SOX4, KAT5 and HDAC1**

**Supplementary Table 2. PCR primer list**
