# Supplementary material for: KAT5-mediated SOX4 acetylation orchestrates chromatin remodeling during myoblast differentiation
Source: Cell Death Dis. 2015 Aug 20;6(8):e1857–. doi: 10.1038/cddis.2015.190 (PMC4558493; doi:10.1038/cddis.2015.190)

Supplementary Fig. 1

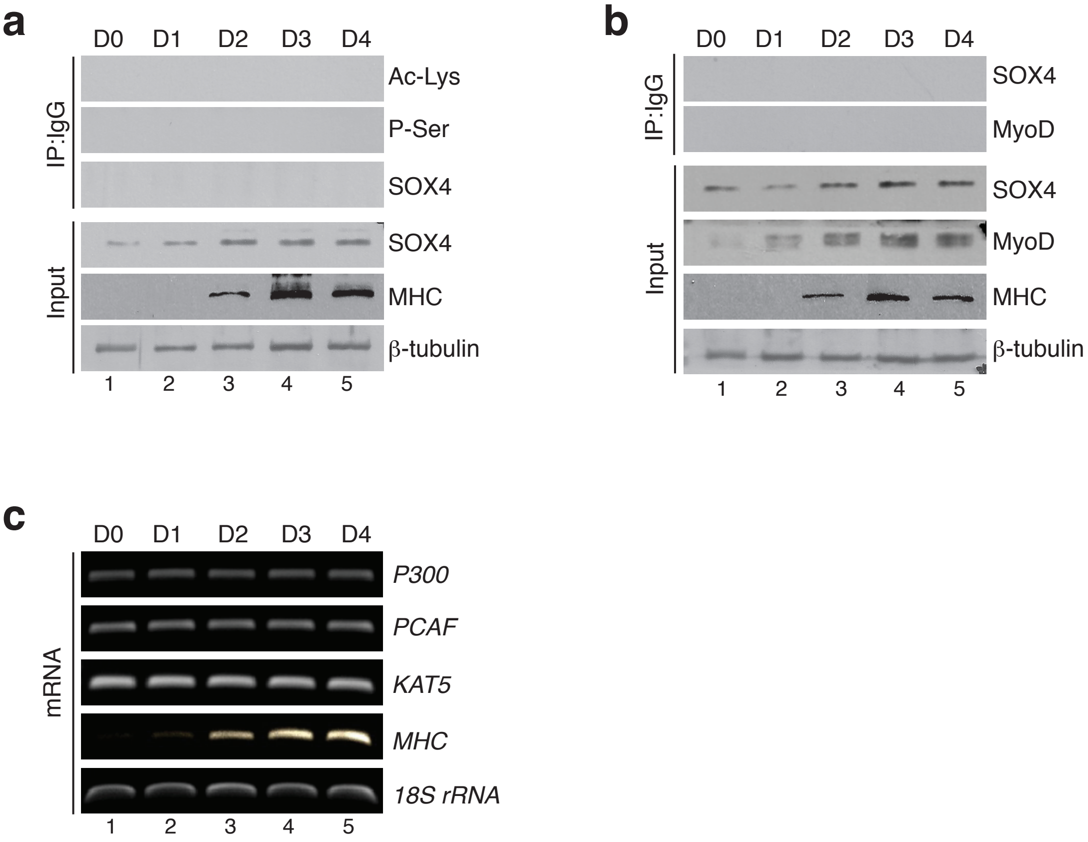

Supplementary Fig. 2

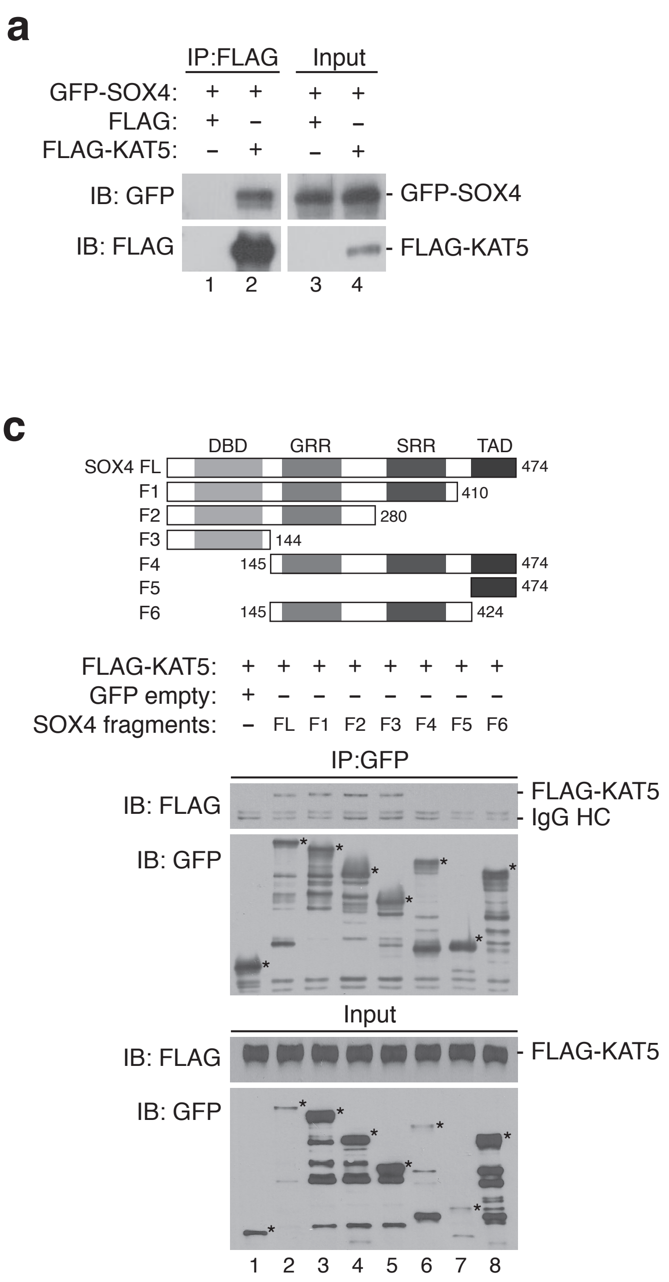

Supplementary Fig. 3

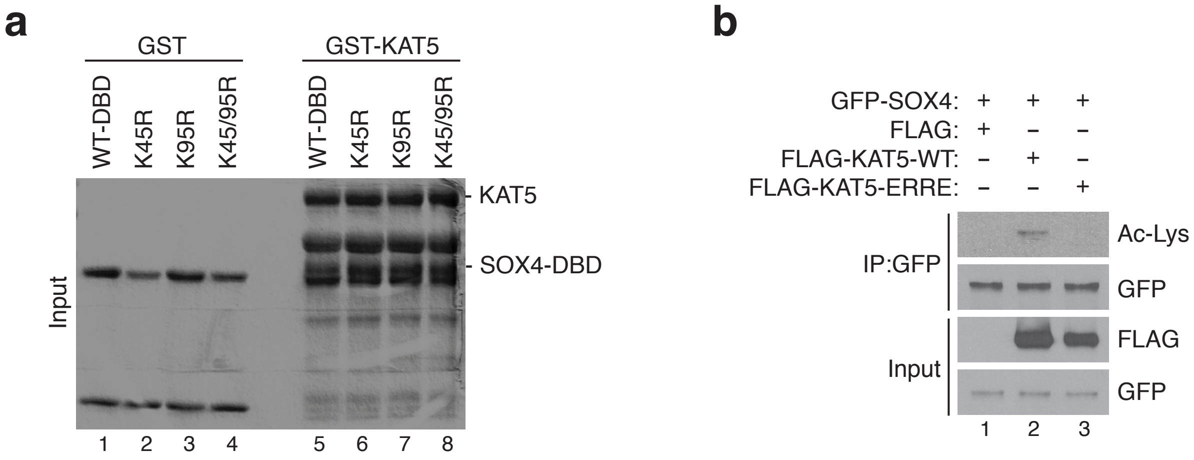

Supplementary Fig. 4

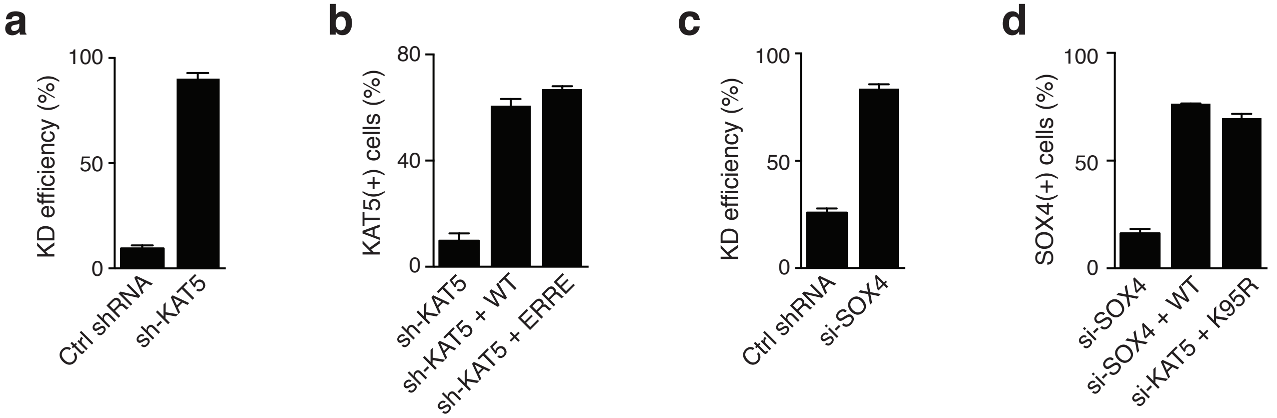

Supplementary Fig. 5

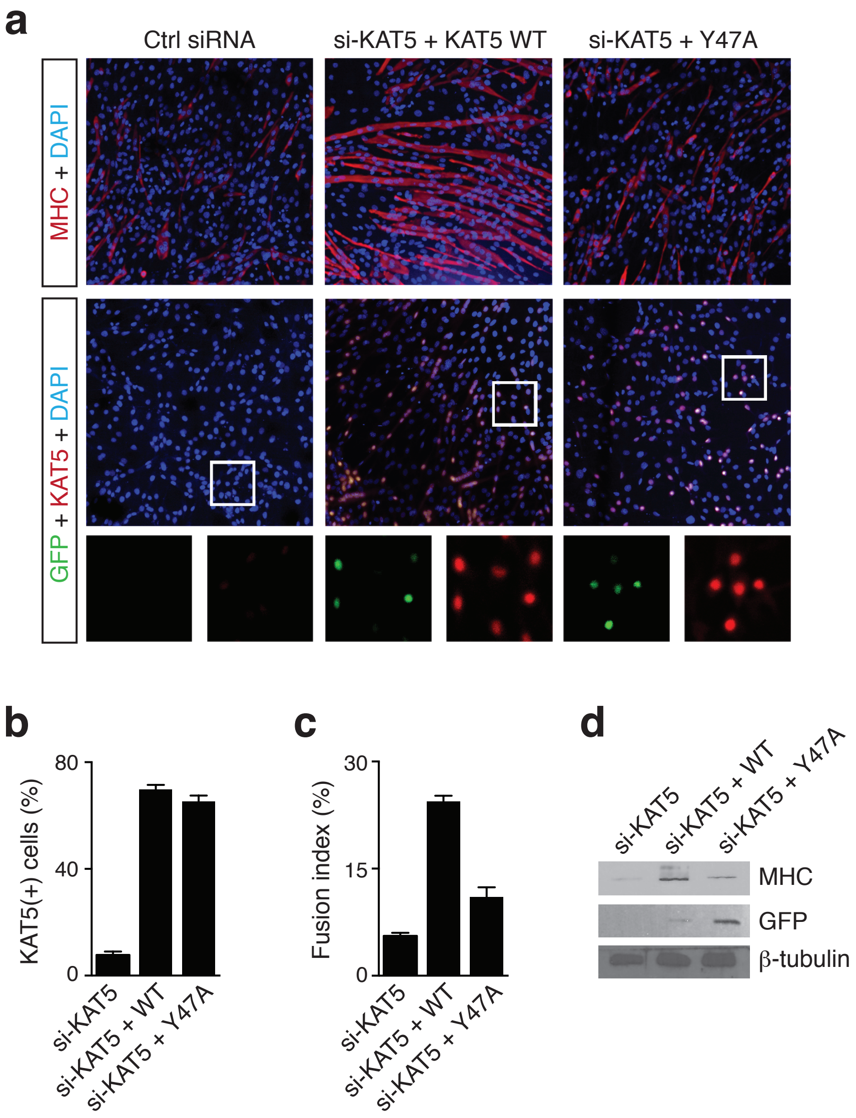

Supplementary Fig. 6

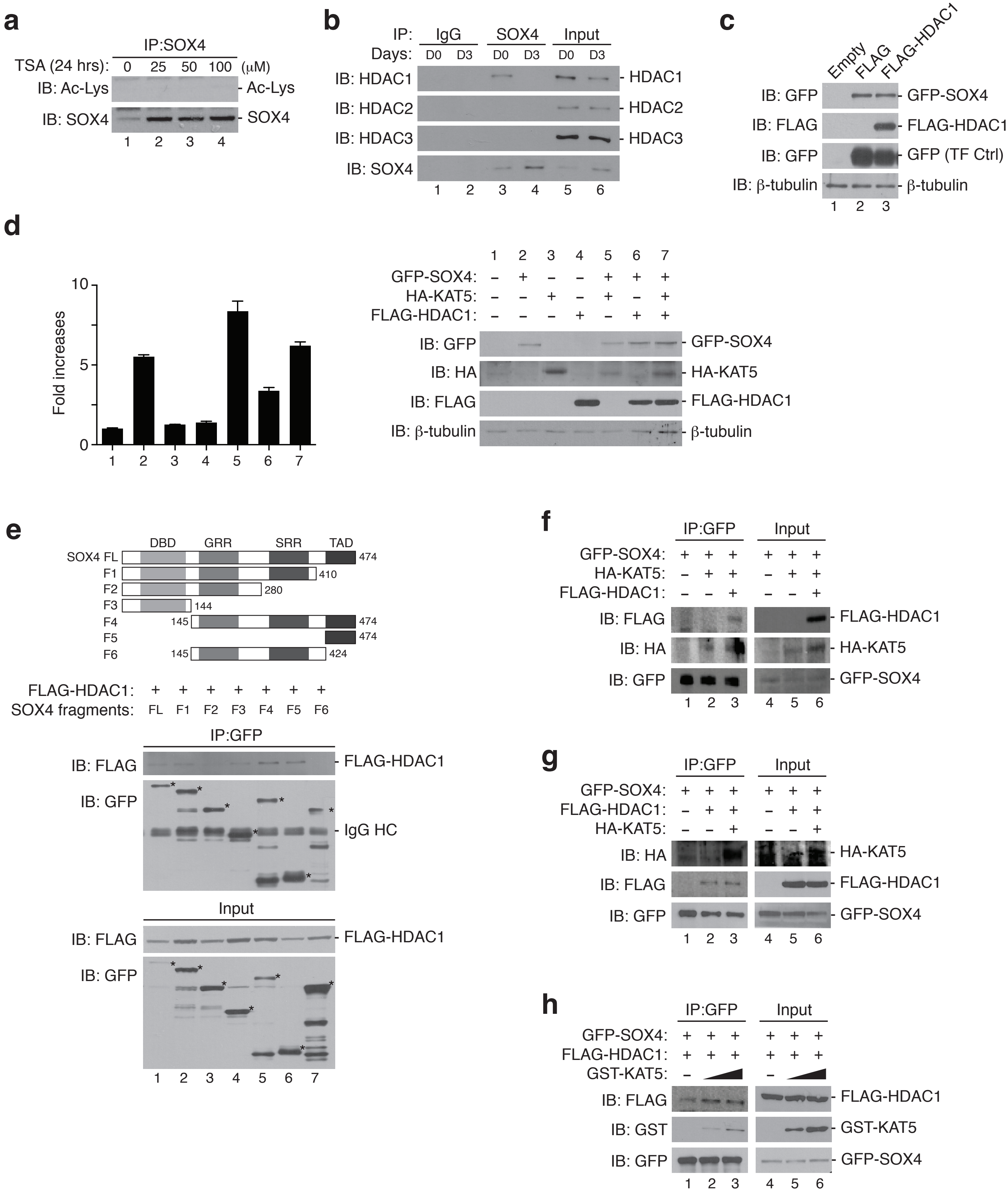

Supplementary Fig. 7

a

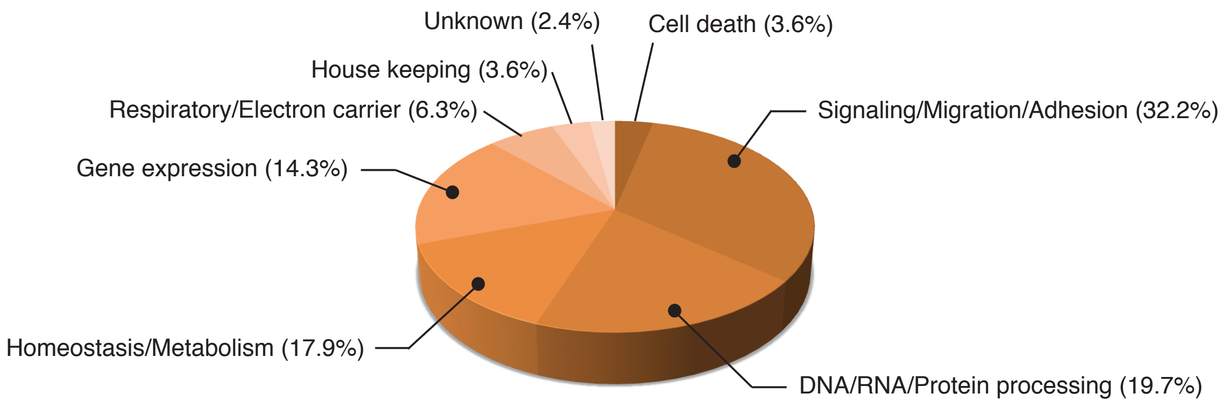

b

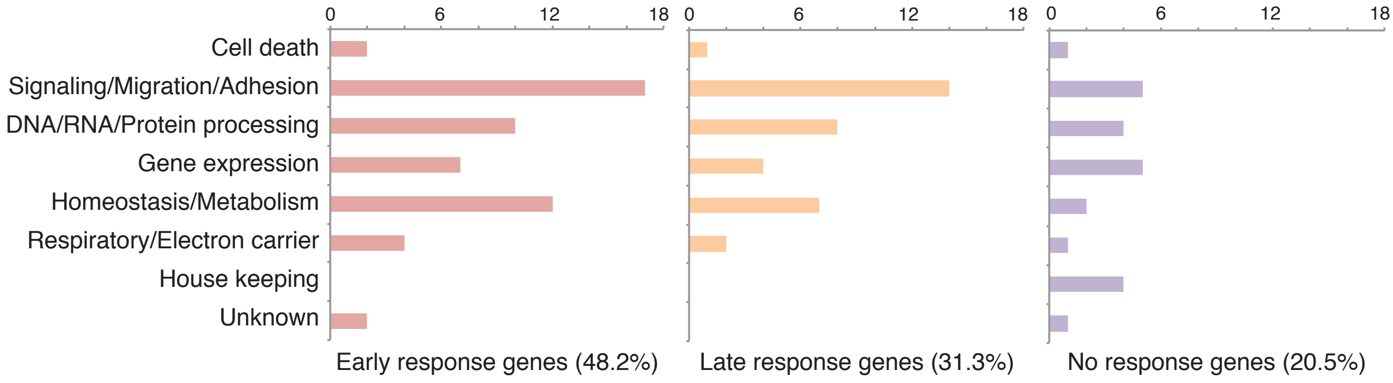

Supplementary Fig. 8

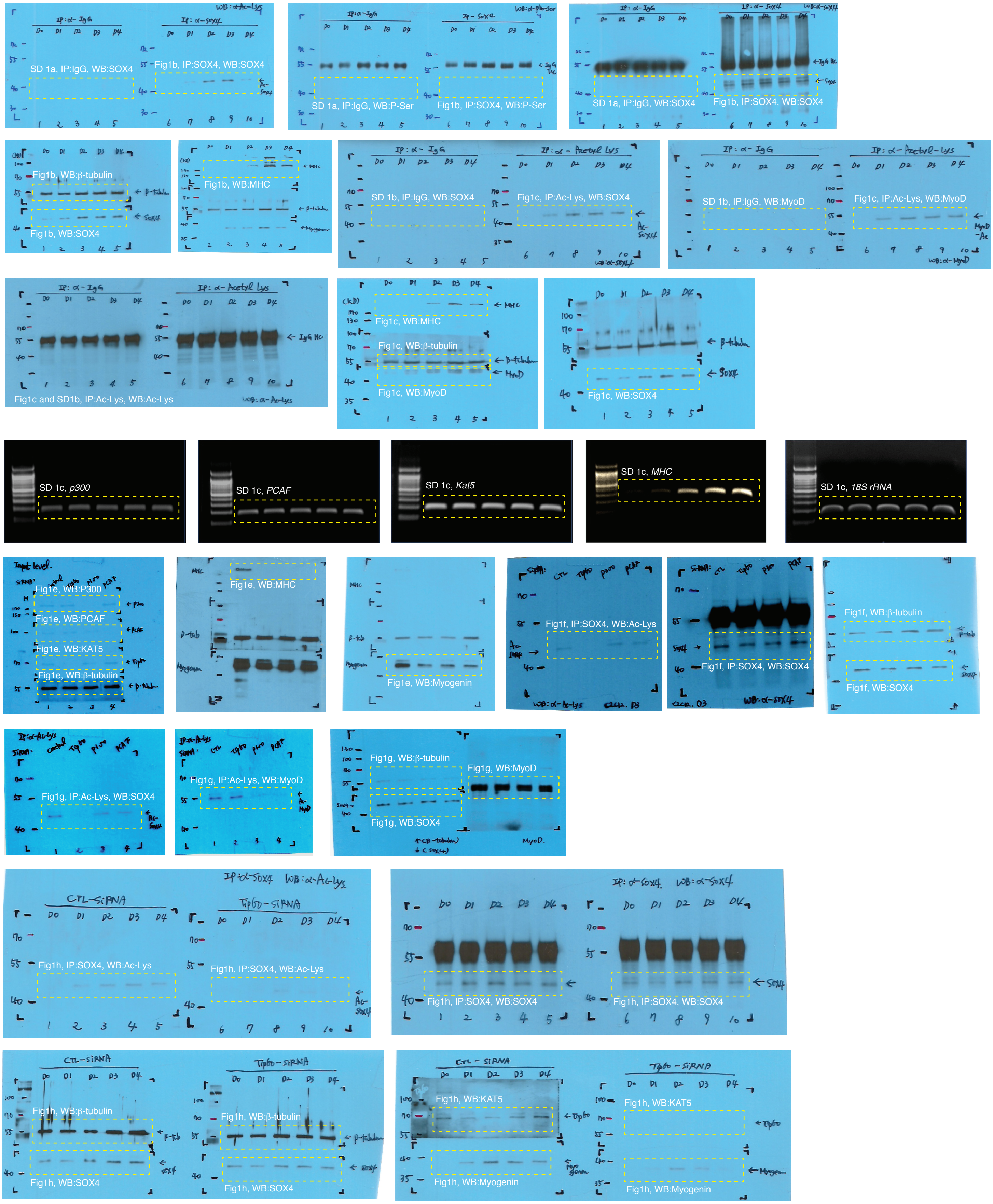

Supplementary Fig. 9

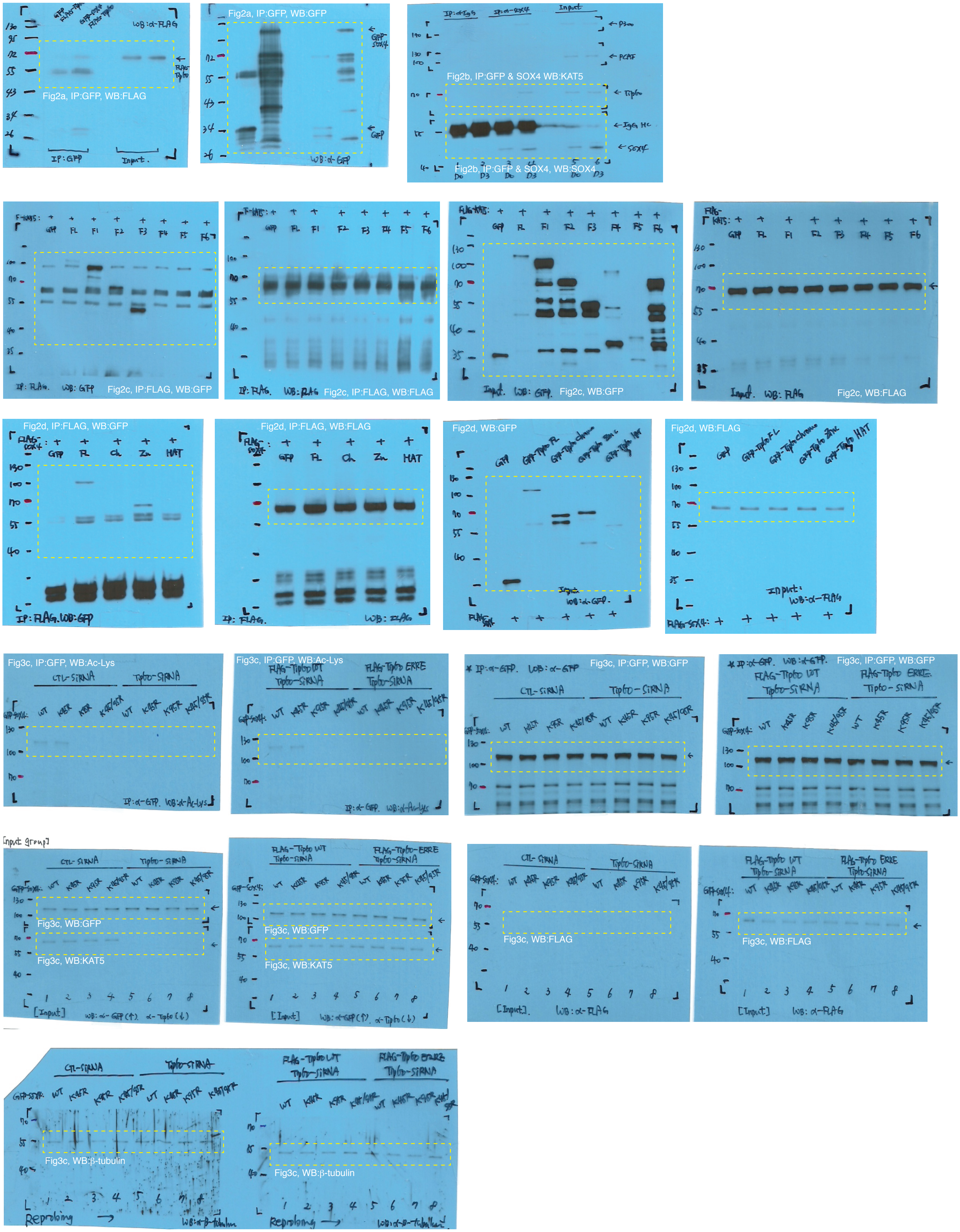

Supplementary Fig. 10

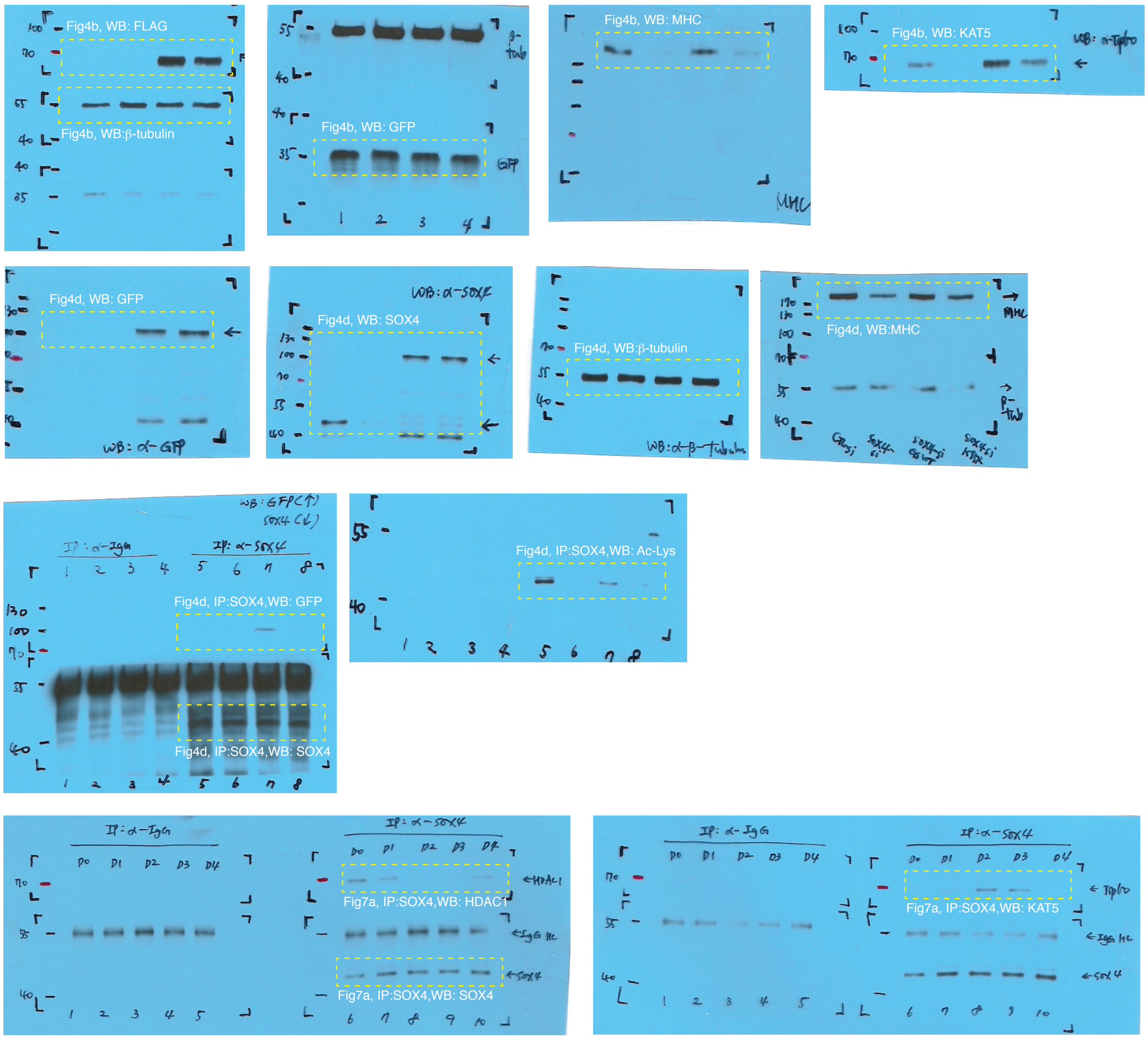

Supplement: Supplementary Figures [file cddis2015190x2.pdf]
